# Biodegradation of Alprazolam in Pharmaceutical Wastewater Using Mesoporous Nanoparticles-Adhered *Pseudomonas stutzeri*

**DOI:** 10.3390/molecules27010237

**Published:** 2021-12-31

**Authors:** Mahdi Shahriarinour, Faten Divsar, Fereshteh Kamalpour Dahka, Sharareh Nezamivand Chegini, Mohamad Mahani, Arash Moeini, Pierfrancesco Cerruti

**Affiliations:** 1 Department of Microbiology, Rasht Branch, Islamic Azad University, Rasht 4147654919, Iran; m.shahriarinour@gmail.com (M.S.); asal.kp89@yahoo.com (F.K.D.); moonsara60@gmail.com (S.N.C.); 2 Department of Chemistry, Payame Noor University, Tehran 193953697, Iran; 3 Department of Chemistry, Faculty of Chemistry and Chemical Engineering, Graduate University of Advanced Technology, Kerman 7631818356, Iran; mahani@kgut.ac.ir; 4 School of Life Sciences Weihenstephan, Technical University of Munich, 85354 Freising, Germany; arash.moeini@tum.de; 5 Institute for Polymers, Composites and Biomaterials (IPCB-CNR), Via Campi, Flegrei 34, 80078 Pozzuoli, Italy

**Keywords:** alprazolam, biodegradation, mesoporous nanoparticles, pharmaceutical industry wastewater, water remediation, *Pseudomonas stutzeri*

## Abstract

The release of pharmaceutical wastewaters in the environment is of great concern due to the presence of persistent organic pollutants with toxic effects on environment and human health. Treatment of these wastewaters with microorganisms has gained increasing attention, as they can efficiently biodegrade and remove contaminants from the aqueous environments. In this respect, bacterial immobilization with inorganic nanoparticles provides a number of advantages, in terms of ease of processing, increased concentration of the pollutant in proximity of the cell surface, and long-term reusability. In the present study, MCM-41 mesoporous silica nanoparticles (MSN) were immobilized on a selected bacterial strain to remove alprazolam, a persistent pharmaceutical compound, from contaminated water. First, biodegrading microorganisms were collected from pharmaceutical wastewater, and *Pseudomonas stutzeri* was isolated as a bacterial strain showing high ability to tolerate and consume alprazolam as the only source for carbon and energy. Then, the ability of MSN-adhered *Pseudomonas stutzeri* bacteria was assessed to biodegrade alprazolam using quantitative HPLC analysis. The results indicated that after 20 days in optimum conditions, MSN-adhered bacterial cells achieved 96% biodegradation efficiency in comparison to the 87% biodegradation ability of *Pseudomonas stutzeri* freely suspended cells. Kinetic study showed that the degradation process obeys a first order reaction. In addition, the kinetic constants for the MSN-adhered bacteria were higher than those of the bacteria alone.

## 1. Introduction

Pharmaceutical contaminants were first identified in aqueous environments during 1970s [1]. The presence of toxic and nonbiodegradable pharmaceutical compounds in industrial, agricultural, and pharmaceutical wastewaters is considered as a relevant source of water pollution [2]. These persistent organic pollutants (POPs) can show adverse biological effects on aquatic organisms [3]; therefore, their removal plays a key role in controlling water contamination [4].

Several suitable methods have been reported to remove POPs from water, including adsorption on various sorbents, chemical decomposition by oxidation or photodegradation, and biodegradation with microbiological treatments [5,6]. In particular, biodegradation technologies are utilized as a good alternative to traditional physicochemical methods due to low cost and environmental friendliness. Additionally, microorganism immobilization can result in ease of processing and long-term reusability, which reduce the overall cost [7]. Aissaoui et al. developed a bacterial consortium, formed by four bacterial strains belonging to genera known to be good biodegraders for biological removal of the diclofenac, ibuprofen, and sulfamethoxazole [8]. Similar results were reported by Yang et al. (2020) from a study in which four antibiotic-degrading bacterial strains, SF1 (*Pseudomonas* sp.), A12 (P*seudomonas* sp.), strains B (*Bacillus* sp.), and SANA (*Clostridium* sp.), were isolated, identified, and tested under aerobic and anaerobic conditions for degradation of oxytetracycline, tetracycline, chlortetracycline, amoxicillin, sulfamethazine, sulfamethoxazole, and sulfadimethoxine in sludge [9].

Additionally, nanotechnology is being explored as a promising tool that has demonstrated remarkable accomplishments in various fields including wastewater treatment. Nanostructures offer unparalleled opportunities to make more effective catalysts and redox active media for wastewater purification, owing to their small size, large surface area, and ease of functionalization [10]. The combined use of nanomaterial and bioremediation practices enhances their physical, chemical and biological interactions both in soil and water [11]. Moreover, possible synergistic effects are beneficial to improve pollutant removal efficiency. Sarioglu et al. immobilized two different sodium dodecyl sulfate (SDS) biodegrading bacterial strains onto cellulose acetate nanofibers to obtain reusable materials for surfactant remediation in aqueous systems [12]. In another case, *Rhodobacter capsulatus* bacteria were immobilized onto iron oxide–biochar nanocomposites and used as effective material for wastewater bioremediation. In this composite, iron oxide nanoparticles (NPs) were used for catalysis and magnetic separation, bacteria for biodegradation, and biochar for increased adsorption of the pollutant [13]. Khatoon et al. revealed that α-Fe_2_O_3_ magnetic NPs could be applied as a potential carrier in *Bacilli* cell immobilization and biodegradation of atrazine herbicide with great efficiency [14]. Furthermore, a CdS quantum dots–*Geobacter sulfurreducens* biohybrid demonstrated great ability in light-driven degradation of methyl orange [15].

Among pharmaceutical compounds, alprazolam, an antidepressant agent, is one of the main compounds in the pharmaceutical industries and a common environmental POP with hazardous properties for humans and animals [16]. Alprazolam contamination of water puts live organisms at great risk because it can impact the function of eukaryotic cells [17]. Alprazolam can enter the environment through contaminated surface waters during its production. Furthermore, its existence in groundwater resources and return to the water cycle can be considered a threat to the lives of humans, animals, plants, and other ecosystem elements. Romeiro et al. studied alprazolam removal from aqueous solutions through heterogeneous photocatalysis and reported the main factors influencing catalytic process [18]. They found that mesoporous TiO_2_ system can be applied for eliminating alprazolam due to the photocatalytic effect of TiO_2_ related to hydroxyl radicals. However, to the best of our knowledge, biodegradation of alprazolam by microorganisms has never been evaluated. In the present study, we report the removal of alprazolam from contaminated water by MCM-41 mesoporous silica nanoparticle (MSN)-adhered *Pseudomonas stutzeri* bacteria. MSNs feature large loading capacity, biocompatibility, ease of production, and tunable pore diameters and volumes [19]. Therefore, bacterial immobilization on drug-loaded MSN particles allows enhanced delivery of drugs to the adhered bacteria, thereby facilitating drug removal. Herein, an alprazolam-resistant bacterial strain (*Pseudomonas stutzeri*) in pharmaceutical industry wastewaters is first selected and then attached to MSN. Afterward, the ability of the MSN-adhered bacteria to eliminate alprazolam from wastewater is assessed, compared to suspended bacterium culture and bare MSN.

## 2. Results and Discussion

### 2.1. Isolation and Screening of Alprazolam-Degrading Bacteria

**Figure 1:**
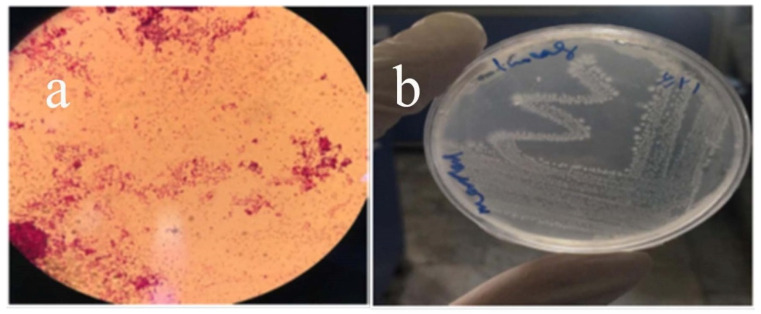
(**a**) The image related to the stained slide of the alprazolam-removing bacterium isolated from pharmaceutical wastewater. (**b**) Growth of isolated bacterium coded as PDS1 in BSM medium at 37 °C.

Four bacterial strains were isolated from pharmaceutical wastewater. Based on the results of inoculating isolated *Pseudomonas stutzeri* on BSM, only one strain, coded as PDS1, can grow in the alprazolam-containing medium as the only source for carbon and energy (Figure 1). PDS1 were Gram-negative rods, which were responsible for a purple pigment color in EMB agar. The main physiological characteristics of PDS1 were: Gram-negative, motile, oxidase-positive, urease, and positive ONPG. Table 1 summarizes the biochemical characteristics of PDS1 strain.

### 2.2. Molecular Identification of the Isolated Strain

**Figure 2:**
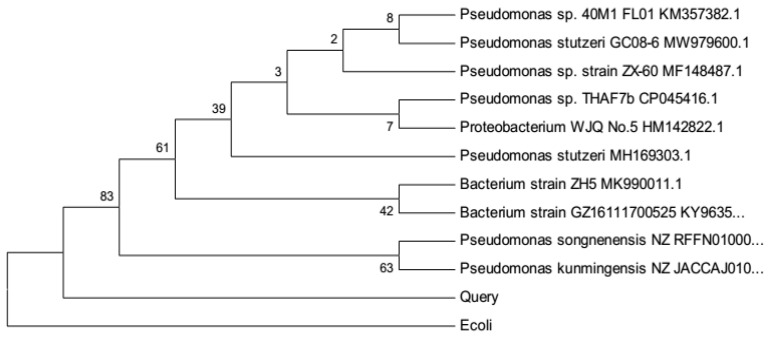
Representation of the phylogenetic tree of isolated strain from gene bank sequencing.

The isolated strain was molecularly identified by amplifying and sequencing the 16SrRNA gene and, consequently, comparing with the known 16SrRNA sequence databases. The molecular tree obtained in this study was based on 16SrRNA sequences. The neighbor-joining distance tree was rendered in the Figure 2 with a similar phylogenetic relationship within and between Pseudomonas and its similarity genes from 16SrRNA phylogeny. In this analysis, E. coli gene was selected as an outgroup taxon. Phylogenetic analysis of the 16SrRNA data set by the neighbor-joining method verified that sequencing belongs to two monophyletic clades (with bootstrap values of 83 or 63%). Based on the results, our sequence has similarities with two closely related species including Pseudomonas songnenensis and Pseudomonas kunmingensis, forming a highly supported monophyletic group with bootstrap values of 63%. However, as shown in Figure 2, the main group is a larger assemblage, as it consists of five well-supported clades including Pseudomonas, Bacterium, and proteobacterium in the form of polyatomic, with 83% similarity to our sequence.

### 2.3. Characterization of MSN

**Figure 3:**
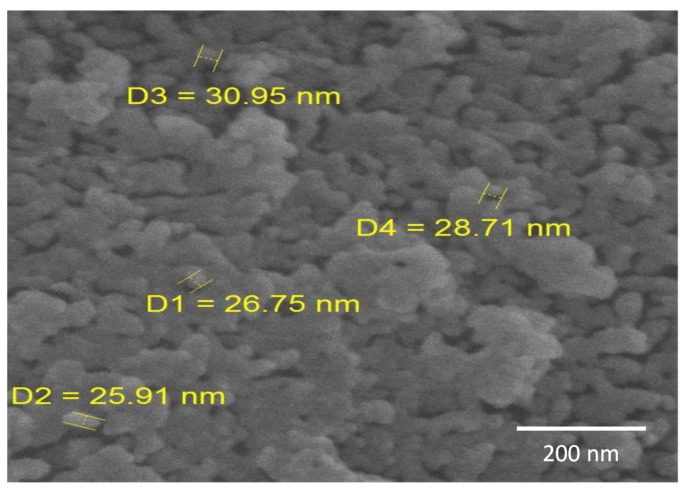
SEM image of the amine-modified MSN.

Figure 3 displays the scanning electron microscopy (SEM) image of the synthesized amine-modified MSN. As shown, the primary silica particles are uniform and spherical, with a mean size of 28 nm, and they partly agglomerate, forming aggregates in the range of hundreds of nm.

**Figure 4:**
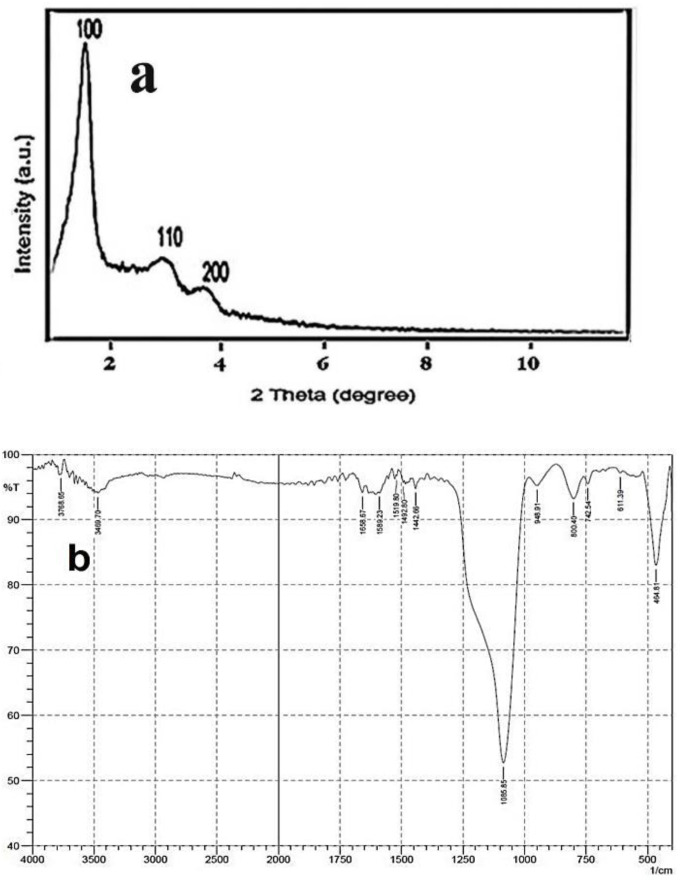
(**a**) XRD pattern and (**b**) FTIR spectrum of the amine-modified MSN.

Powder X-ray diffraction pattern of amine-modified MSN is shown in Figure 4a. The sample showed a typical mesoporous structure with three sharp peaks corresponding to Miller indices (100), (110), and (200). The prominent peak at 2theta ranging between 1° and 2° corresponds to (100) plane, which indicates an MCM-41 MSN structure. Figure 4b shows the FTIR analysis of amine-modified MSN. The bending modes of the amino groups chemisorbed into channels of MSN are observed at 1659 and 1589 cm^−1^. The bands between 3400 and 3600 cm^−1^ indicate N-H stretching vibration (overlap with O-H at signal), and absorptions at 1025–1300 cm^−1^ are ascribed to the C-N linkages of amino groups on the surface of MSN.

**Figure 5:**
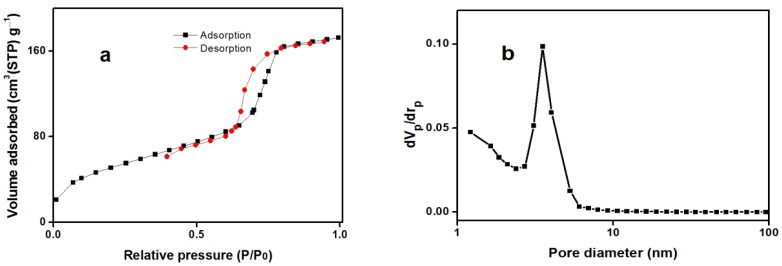
(**a**) Nitrogen adsorption/desorption isotherms and (**b**) BJH pore size distribution of the amino-modified MSN.

Figure 5a,b displays the nitrogen adsorption/desorption isotherms and pore size distributions of the prepared sample, respectively. It seems that MSN sample exhibits the type IV isotherm with a distinct capillary condensation step, which is a characteristic pattern of mesoporous materials according to the classification of the IUPAC. The data of the BET specific area, the BJH pore volume, and the BJH pore diameter were 150.75 (m^2^/g), 0.25 (mL/g), and 6.15 (nm), respectively.

**Figure 6:**
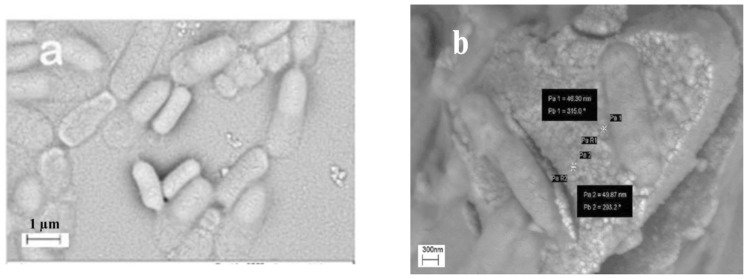
SEM images related to (**a**) the free bacteria and (**b**) MSN-adhered bacteria.

SEM images related to the free (Figure 6a) and MSN-adhered bacteria (Figure 6b) show the accumulation of MSN particles around the bacterium and their linkage to the bacterial cell walls due to the particle surface modification with amino groups. Indeed, at physiological pH of 7.4, amino groups (with pk_a_ 9.7) are mostly protonated, forming positively charged NH_3_^+^ ions on the mesoporous nanoparticles. Therefore, the electrostatic interactions contribute to the high affinity between the negatively charged bacterium surface and positively charged amine-modified MSN [20]. Indeed, the value of the zeta potential measurement of MSN was negative, while it increased to positive after amine functionalization [21]. This is because the silanol groups were negatively charged under a wide range of pH conditions, while the aminopropyl groups attached on MSN were positively charged when suspended in water.

### 2.4. Effect of Alprazolam Concentration and pH on Bacterial Growth and Alprazolam Biodegradation

**Figure 7:**
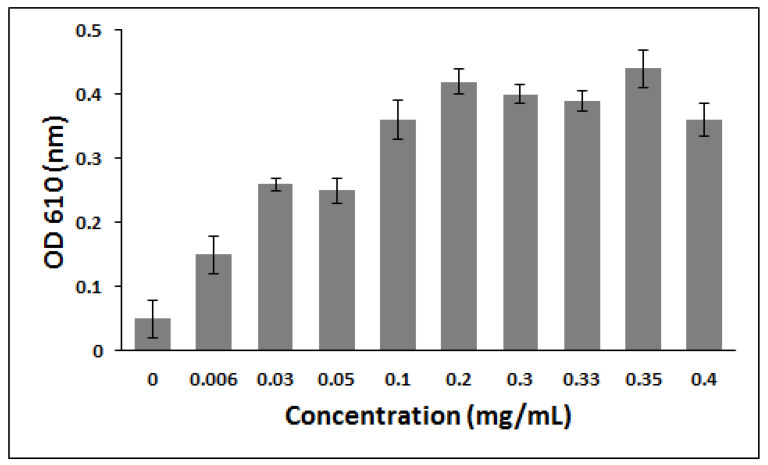
The effect of alprazolam concentration on the OD of *Pseudomonas stutzeri* culture after three days of cultivation. The data are mean absorbance of three independent experiments, and the error bars represent the standard deviation of the mean.

In a first series of experiments, the effect of alprazolam concentration on Psudomonas Stutzeri was evaluated. The isolated strain was inoculated in the TSA medium containing up to 0.4 mg/mL concentration of alprazolam, and the number of bacterial cells was determined after three days through UV–Vis spectrophotometry measurement at λ_max_ 610 nm. It was noticed that bacterial growth was affected by alprazolam concentration, as OD increased by increasing alprazolam concentration in TSA medium up to 0.2 mg/mL and then decreased slightly (Figure 7). The decrease of bacterial cell number at higher concentrations of alprazolam might be attributed to the toxicity of the drug to bacterial cells through the inhibition of metabolic activity, as well as to saturation of the cells with drug byproducts [22].

The ability of free suspended Pseudomonas stutzeri cultures to biodegrade alprazolam at the initial concentration of 0.1 mg/mL was assessed at different pH values. One day after inoculation, the final concentrations of alprazolam were 0.099, 0.094, and 0.097 mg/mL at pH values of 5.4, 7.4, and 8.5, respectively. In addition, at the same pH values, the final concentration at 20 d after inoculation decreased to 0.066, 0.013, and 0.055 mg/mL. It is worth noting that, in the absence of bacterial cells and MSN, no degradation of alprazolam was observed.

**Figure 8:**
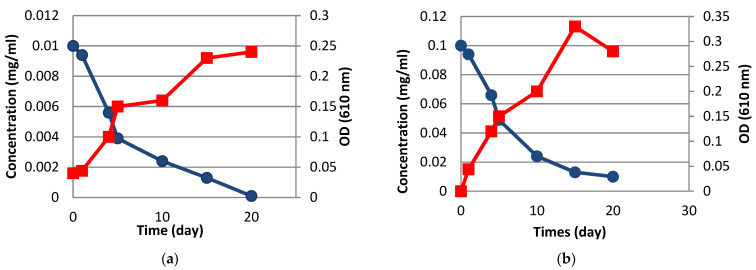
Alprazolam degradation and growth of *Pseudomonas stutzeri* in TSA containing different concentrations of alprazolam (pH 7.4); (**a**) 0.01 mg/mL, (**b**) 0.1 mg/mL. (-●-) alprazolam concentration, (-■-) bacterial growth.

Figure 8 displays the degradation kinetics at pH 7.4 related to two concentrations of alprazolam, as well as bacterial growth rate, which represents the ability of the isolated strain to utilize alprazolam as a carbon and energy source. The isolated Pseudomonas stutzeri strain was able to remove alprazolam at 0.01 and 0.1 mg/mL concentrations over the 20-day experiment with 99 and 87% efficiencies, respectively. It was noted that an increase in the concentration of alprazolam caused the rate of its degradation to decrease, while the cell log phase was longer (Figure 8). In comparison, no significant degradation was observed for alprazolam in the negative control medium, confirming the high degradation efficiency of the isolated strain. In addition, bacterial growth in the TSA with 0.01 mg/mL alprazolam peaked on the 20^th^ day (O_610_ = 0.24), while the maximum of bacterial growth in the medium containing 0.1 mg/mL alprazolam was observed at 15^th^ day (O_610_ = 0.33) due to higher energy source, then decreased likely due to the growth-inhibiting effect of drug byproducts.

### 2.5. Biodegradation of Alprazolam by the MSN-Adhered Bacteria

The biodegradation efficiency of the bacterial cells modified with different amounts of MSN was studied using a solution of 0.1 mg/mL alprazolam. After 20 days, bacterial cells modified with 5, 10, and 20 mg/mL of MCM-41 MSN yielded biodegradation efficiencies of 66%, 96%, and 86%, respectively. In fact, the alprazolam dissolved in the solution was absorbed by the mesoporous nanoparticles and made more available for the bacterial cells, and then the rate of the biodegradation process increased. Accordingly, the optimum amount of MSN for immobilizing bacteria was determined as 10 mg/mL. Higher amounts of MSN led to increased bacterial mortality and reduced biodegradation efficiencies.

**Figure 9:**
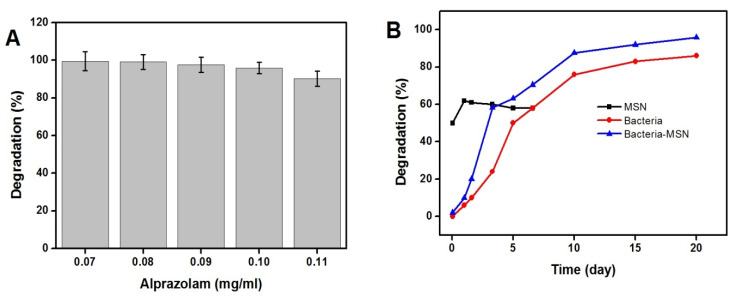
Biodegradation of (**A**) 0.07, 0.08, 0.09, 0.10, and 0.11 mg/mL alprazolam by the MSN-adhered bacteria after 20 days, and (**B**) 0.10 mg/mL alprazolam by MSN (■), freely suspended *Pseudomonas stutzeri bacteria* (●), and MSN-adhered bacteria (▲) throughout 20 days.

Afterward, the 10 mg/mL MSN-modified P. stutzeri sample was added into BSM containing 0.07–0.11 mg/mL alprazolam. As shown in Figure 9A, the MSN-adhered bacteria can easily eliminate up to 0.10 mg/mL alprazolam with 96% efficiency throughout 20 days. However, biodegradation efficiency decreased slightly to 90% for alprazolam concentration of 0.11 mg/mL. MSNs allow alprazolam to stay in the vicinity of bacterial cell so that it can be removed from wastewater by adsorption and bioremediation simultaneously. However, compared to pure MSN, in the case of the MSN–bacterial cell hybrid, a lower number of adsorption sites are available at the very beginning because of the interaction of MSN with the bacterial cells. It is worth noting that pure MSNs remove the drug from the wastewater only by adsorption, without drug destruction. In contrast, the bioremediation process includes degradation and conversion of pollutants to less toxic forms.

Further, the efficiency of the MSN-adhered bacteria in biodegrading 0.1 mg/mL alprazolam across 20 days was compared with that of neat MSN and untreated bacteria. Based on the results in Figure 9B, MSN was able to efficiently remove alprazolam from the medium through adsorption, with 60% removal efficiency in the first two hours. The amount of alprazolam removed by biodegradation steadily increased, being lower in the first 5 days, then reaching 87% after 20 days. However, the ability of the MSN-adhered bacteria to biodegrade alprazolam over 20 days was constantly higher than that of bacterium alone, which can be related to simultaneous adsorption and biodegradation of the drug. In other words, MSN increased the local concentration of alprazolam around the bacteria through adsorption and, thus, enhanced the biodegradation efficiency. This result is in accordance with a previous report showing the increasing drug delivery activity with nanosized particles [23,24].

### 2.6. Kinetic Modeling

From the kinetic curves reported in Figure 9B, the kinetic parameters (reaction rate constant and reaction rate) were calculated by fitting the data with first and second order kinetic models. The linearized form of the first and second order kinetic models of alprazolam biodegradation are as follows:

−ln(C/C_0_) = k_1_t (1)

(1/C) – (1/C_0_) = k_2_t (2)

where C_0_ and C (mg/mL) indicate the initial and final concentrations of the drug, respectively. K_1_ (day^−1^) and k_2_ (mL/mg day) are considered the rate constants of the first and second order kinetic models, respectively. The plot −Ln (C_0_/C) versus t should give a straight line when the first order kinetics is obeyed, while when a second order kinetics is followed, the plot [(1/C) – (1/C_0_)] versus t should give a straight line. K_1_ and k_2_ values can be specified from the slope of the plots.

**Figure 10:**
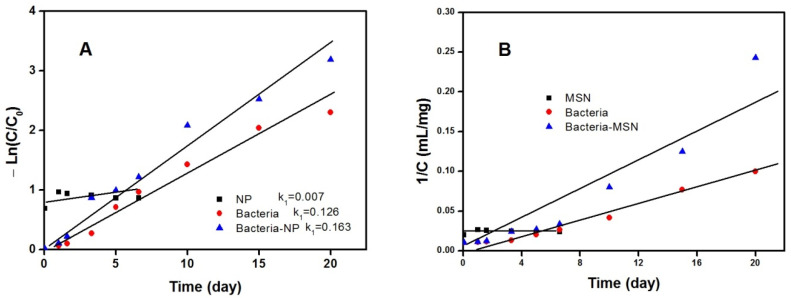
First (**A**) and second (**B**) order kinetics of alprazolam degradation during 20 days for MSN, freely suspended *P. stutzeri* bacteria, and MSN-adhered bacteria at T = 298 K. C_0_ = 0.1 mg/mL and pH = 7.4.

As shown in Figure 10 and Table 2, regression values of first order models were more reliable compared to those of the second order. Additionally, the kinetic constant k_1_ value for the MSN-adhered bacteria was 0.1628 day^−1^, compared to 0.1263 day^−1^ for the bacteria alone. That is, the kinetic constant value for degrading alprazolam increased by about 30% in the presence of MSN compared to the freely suspended culture. It is supposed that MSN, thanks to its high surface area, can increase the concentration of alprazolam in proximity of the bacterial surface. In addition, the microenvironment change after immobilization can trigger modifications in metabolic activity, cell morphology, and physiology [25].

### 2.7. Thermodynamic Study

The effect of the temperature on the alprazolam degradation is another important parameter to account for in the optimization of the wastewater treatment conditions. The Eyring–Polanyi equation represents the rate change of a chemical process with temperature. The linear form of this equation is as follows:

ln[(k.h)/(K_B_T)] = −(∆H/R)1/T + ∆S/R

where k indicates the rate constant, K_B_ is the Boltzmann constant = 1.38066 × 10^−23^ J/K, R is the universal gas constant = 8.31441 J/mol K, h represents the Planck constant = 6.6262 × 10^−34^ J s, and ∆H (J/mol) and ∆S (J/mol K) are regarded as the changes of enthalpy and entropy, respectively. ∆H and ∆S can be calculated from the slope and intercept of the fitting line against the reciprocal temperature.

On the other hand, the change in Gibbs free energy (∆G) of the process can be obtained from the van’t Hoff equation after determining ∆H and ∆S values:

∆G = ∆H − T∆S

**Figure 11:**
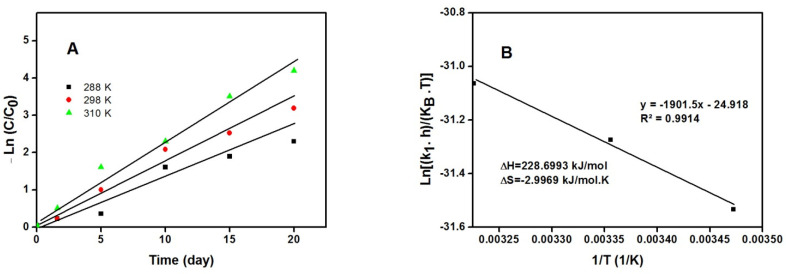
(**A**) First order kinetic model for degradation of alprazolam at 288, 298, 310 K and pH = 7.4 across 20 days. (**B**) Eyring–Polanyi model for degradation of alprazolam at different temperatures and pH = 7.4 across 20 days to determine thermodynamic parameters.

The test were carried out in a concentration of alprazolam C_o_ = 0.1 mg/mL, pH = 7.4, and three different temperatures (288, 298, 310 K). As shown in Figure 11A, the k_1_ values were 0.1211, 0.1625, and 0.2072 day^−1^ at 288, 298, and 310 K, respectively. As illustrated in Figure 11B, using the Eyring–Polanyi equation, ∆H and ∆S values of this process were +288.7 kJ/mol and −3.0 kJ/mol K, respectively. The positive ∆H value accounts for the endothermic character of the process, and the negative value of ∆S represents the decreasing disorder at the interface between alprazolam and the degrading agent. Thus, ∆G values at 288, 298, and 310 K were 1091. 8, 1121.8, and 1151.8 kJ/mol, respectively. The positive value of ∆G thermodynamically indicates that the process is nonspontaneous.

## 3. Materials and Methods

All chemicals were of analytical grade. Thus, they were used without further purification. Metal salts, methanol, hydrochloric acid, and sodium hydroxide were purchased from Merck (Merck Co., Darmstadt, Germany). Additionally, cetyltrimethylammonium bromide (CTMABr), tetraethyl orthosilicate (TEOS), and Pluronic P123 (EO20PO70EO20, Mav = 5800) were provided by Sigma–Aldrich (Sigma–Aldrich Co, Darmstadt, Germany). Double distilled water was employed to prepare aqueous solutions.

The characterization studies were performed by X-Ray diffraction (XRD) employing a Philips X’PertPro diffractometer using Cu_Ka_ radiation (operating at 40 kV and 40 mA); UV–Vis spectroscopy (PerkinElmer Lambda 25 spectrophotometer); Fourier transform infrared (FTIR) spectra (Shimadzu-8900 spectrometer); field emission scanning electron microscopy (FESEM) using a TESCAN electron microscope operating at 30 kV; and specific surface area (BET) measurement employing a BELSORP mini II system. This equipment was also utilized to calculate the pore distribution by the BJH method.

### 3.1. Preparation of Alprazolam Stock Solution

Alprazolam was dissolved in methanol and made up to volume with double distilled water in order to prepare 100 mg/mL stock solution. Then, the solution was stored at 4 °C. Working solutions were daily produced by diluting the stock solutions appropriately using doubled distilled water.

### 3.2. Sampling and Isolation of Bacteria

Regarding the isolation of bacterial strains, the samples were collected from the 5–50 cm depth of the wastewater container in the pharmaceutical company in 100 mL sterile bottles and transferred to the laboratory immediately. In addition, pharmaceutical wastewater samples were passed through a 0.45 syringe filter, added into tryptic soya agar (TSA), and incubated at 22 °C for 72 h. Then, each medium with obvious turbidity was inoculated in R2A, blood agar, and eosin methylene blue (EMB) agar in order to isolate bacterial strains. R2A is considered as a reduced culture medium, the use and long-term incubation at low temperatures of which result in improving the recovery of the bacteria damaged by chlorine stress in treated waters, leading to an increase in bacterial number.

### 3.3. Selecting Alprazolam-Degrading Bacteria

Basal salt medium (BSM) was applied for assessing the ability of isolates to degrade alprazolam. After obtaining the pure culture, BSM containing 5 g/L NH_4_Cl, 0.1 g/L KH_2_PO_4_, 0.2 g/L MgSO_4_·7H_2_O, 3.78 g/L Na_2_HPO_4_·12H_2_O, 0.01 g/L FeSO_4_·7H_2_O, and 0.02 g/L CaCl_2_·4H_2_O, as well as a 0.1 g/L solution of trace elements including the salts of 0.5 g/L ZnCl_2_·7H_2_O, 0.2 g/L MnSO_4_·H_2_O, 0.5 g/L CuSO_4_·5H_2_O, and also TSA were used as the growth media for the culturing of bacteria. The pH of the culture medium was adjusted to neutral, and alprazolam solution (1% *v*/*v*) was utilized as the only source for supplementary carbon and energy. We used 1 g/L glucose as an inducer for isolation of bacterial strains. The isolated strains were inoculated in BSM and incubated at 22 °C for 15 days at 120 rpm. 

### 3.4. Bacterial Recognition

Bacterial isolate was recognized through morphological and physiological analysis by using Microgen bacterial identification kit (GNA + BID). In addition, they were molecularly identified through amplification and 16SrRNA gene sequencing. Briefly, the bacterium was cultured in TSA overnight and harvested by centrifuging (8000× *g*/10min). Further, bacterial DNA was extracted by using GenAllTM DNA extraction kit (Seoul, South Korea), and 16SrRNA gene segment was amplified by using PCR thermocycler (BioER XP cycler, Zhejiang, China) by mL.

Forward 27F: (5′-AGAGTTTGATYMTGGCTCA-3′)

Reverse 1492R: (5′-CGGTTACCTTGTTACGACTT-3′)

Heating cycle was scheduled as 5 min at 94 °C, 30 min one-cycles at 94 °C, 1 min at 55 °C, 1 min at 72 °C, and 5 min at 72 °C.

PCR solution was sequenced per 16SrRNA (GATC Biotech, Konstanz, Germany), the sequence was sent to the NCBI GenBank website (http://www.ncbi.nlm.nih.gov/BLAST.htm, accessed on 1 August 202l), and other published sequences were stored in the GenBank database. Further analyses were performed by using Bio Edit 7.0 and Clustal W 6.0 software in order to draw the phylogenetic tree.

### 3.5. Effect of Alprazolam Concentration on the Bacterial Growth

The effect of alprazolam poisoning on the bacterial growth was determined before evaluating the potential of the isolated strain for removing the alprazolam. In this regard, triplicate samples of the different concentrations of alprazolam (0.006–0.4 mg/mL) were prepared in the TSA medium. In addition, 100 μL of bacterial suspension with the approximate population of 1.5 × 108 CFU/mL was added into 9.9 mL of TSA medium. Then, the tubes were incubated at 22 °C for 72 h at 120 rpm, and bacterial growth was obtained by measuring the optical density of each medium at 610 nm (HALO DB-20 Dynamica, Salzburg-Mayrwies, Austria).

### 3.6. Assessing the Biodegradation of Alprazolam by Bacteria

The biodegradation of alprazolam was evaluated in two 500-mL Erlenmeyer flasks with 250 mL of the BSM containing 0.01 and 0.1 mg/mL alprazolam. Biodegradation was started by inoculating 5 mL of bacterial suspension (1.5 × 10^8^ CFU/mL) in each flask, while negative control groups received no bacterial cell. Then, the flasks were incubated at 22 °C for 20 days at 120 rpm. The experiment was repeated three times, and the medium was sampled in appropriate intervals for determining bacterial count and alprazolam concentration.

### 3.7. Synthesizing MSN and Its Immobilization on Bacterial Cells

MCM-41 MSN was prepared based on the method mentioned in our previous study [26]. In this regard, 2.9 g of ethylamine (3.33 mL) was mixed with 45 mL of distilled water and stirred for 10 min. Subsequently, 1.48 g of cationic surfactant CTMABr was gradually added along with stirring, and the stirring was continued for 30 min to make a clear solution. Further, 4.57 mL of TEOS as silicon source was dropped into the solution. The final molar ratios in the reaction mixture were obtained as 1.0 SiO_2_:1.66 EA:0.215 CTMABr:125 H_2_O.

In this step, the solution was clear, and its pH was around 12. Then, the pH of the solution was brought to 8.5 by dropwise adding 1 M hydrochloric acid. In addition, a white precipitate appeared in the solution, and the obtained suspension was stirred at room temperature for 2 h to complete the formation of the precipitate. Then, the precipitate was separated by centrifuging, washed with semiwarm water, and finally dried at 45 °C for 12 h.

Regarding the immobilization of MSN on bacterial cells, 5 mL of bacterial suspension (1.5 × 108 CFU/mL) was poured into the flasks with 100 mL sterile BSM containing 5, 10, and 20 mg of MSN. The solutions were stirred by shaker at 120 rpm overnight.

### 3.8. Alprazolam Biodegrading by MSN-Adhered Bacteria

The following procedure was carried out to evaluate the biodegradation of alprazolam by MSN-adhered bacteria. The enriched culture was distributed equally in flasks containing 100 mL of sterile BSM amended with different concentrations of alprazolam as the sole carbon source, ranging from 0.07, 0.08, 0.09, 0.1, and 0.11 mg/mL for organizing test samples. A flask with 100 mL of sterile BSM without alprazolam was used for biotic control. Then, a certain number of MSN-adhered bacteria was added into each flask. After adjusting the pH, the solution was placed on a magnetic stirrer at 1400 rpm/min. Finally, the solution was separated from the adsorbent through centrifugation, and the amount of remaining alprazolam was measured by the HPLC method.

### 3.9. Analysis Method

Bacterial cell population was determined by measuring optical density at 610 nm. After the biodegradation process, the samples were centrifuged at 4 °C for 10 min at 10,000× *g*, and the obtained supernatants were utilized for determining the alprazolam concentration through HPLC analysis with an ultraviolet–visible detector (Waters E2695, Milford, MA 01757 U.S.A.). A Hypersil ODS C18 column (5 µm × 200 mm × 4.6 mm i.d) made of stainless steel was used at ambient temperature. Solvent system consisted of acetonitrile and water (11:9 *v*/*v*), which was used at the flow rate of 1.2 mL/min. The injection volume was equal to 10 μL, and alprazolam was detected at 210 nm.

### 3.10. Statistical Analysis

The numerical data from optical density of each medium were presented by the mean value with the standard deviation. All the experiments were conducted three times, with three replicates used for each experiment. Statistical significance was considered at *p* < 0.05.

## 4. Conclusions

In the present study, we have shown that the *Pseudomonas stutzeri* strain, first isolated from pharmaceutical wastewater samples, could grow with alprazolam as the only source of carbon and energy. The degradation kinetics of alprazolam was determined under different initial concentrations of substrate, as well as pH and temperature. Additionally, since MSNs possess large loading capacity, biocompatibility, and tunable pore diameters and volumes, MCM-41 MSN was allowed to be adsorbed onto the bacterium cells, and it was observed that the resulting biohybrids displayed higher efficiency in removing alprazolam under aerobic conditions. This was due to the high surface area and large loading capacity of MSN, which can increase the available amount of alprazolam in proximity of the bacterial surface. Pure MSNs showed the ability to remove alprazolam from the wastewater by surface adsorption. In contrast, bioremediation neutralized the pollutant by a metabolic process. The kinetic study of alprazolam biodegradation demonstrated that the process obeyed a first order model, and the kinetic constant of the process increased by about 30% in presence of MSN compared to the freely suspended culture. Therefore, MSN-adhered bacterial culture can enhance the potential of *Pseudomonas stutzeri* in bacterial degradation of alprazolam under natural conditions and thermophilic temperatures. In this respect, the assessment of the influence of particle size on biodegradation rate could further enhance the significance of this work.

## Figures and Tables

**Table 1:** Biochemical characteristics of pharmaceutical wastewater-isolated PDS1.

Test	Oxidase	Motility	Nitrate	Urease	Citrate	Indole	H2S	Gram Staining	l-Arabinose	D-Glucose
Result	+	+	+	−	+	−	−	−	−	+

**Table 2:** Kinetic constants of alprazolam degradation in presence of MSN, free, or MSN-adhered *P. stutzeri*.

		Y = ax + b	k1	R2
First order	MSN	y = 0.0072x + 0.8545	0.0072	0.0345
	Bacteria	y = 0.1263x – 0.0011	0.1263	0.9744
	Bacteria–MSN	y = 0.1628x + 0.1171	0.1628	0.9736
Second order	MSN	y = 0.0001x + 0.0237	0.0001	0.0217
	Bacteria	y = 0.0047x + 0.0017	0.0047	0.97
	Bacteria–MSN	y = 0.0109x – 0.0127	0.0109	0.9189

## Data Availability

Not applicable.

## Sample Availability

Not available.
